# Multiple Pharmacotherapy Adaptations for Smoking Cessation Based on Treatment Response in Black Adults Who Smoke: A Randomized Clinical Trial

**DOI:** 10.1001/jamanetworkopen.2023.17895

**Published:** 2023-06-20

**Authors:** Nicole L. Nollen, Jasjit S. Ahluwalia, Matthew S. Mayo, Edward F. Ellerbeck, Eleanor L. S. Leavens, Gary Salzman, Denton Shanks, Jennifer Woodward, K. Allen Greiner, Lisa Sanderson Cox

**Affiliations:** 1 Department of Population Health and the University of Kansas Cancer Center, University of Kansas School of Medicine, Kansas City; 2 Department of Behavioral and Social Sciences, Brown University School of Public Health, Providence, Rhode Island; 3 Department of Biostatistics and Data Science and the University of Kansas Cancer Center, University of Kansas School of Medicine, Kansas City; 4 Department(s) of Internal Medicine, Division of Respiratory and Critical Care, University of Missouri–Kansas City School of Medicine, University Health, Kansas City, Missouri; 5 Department of Family Medicine and Community Health, University of Kansas School of Medicine, Kansas City

## Abstract

**Importance:**

Adapting to different smoking cessation medications when an individual has not stopped smoking has shown promise, but efficacy has not been tested in racial and ethnic minority individuals who smoke and tend to have less success in quitting and bear a disproportionate share of tobacco-related morbidity and mortality.

**Objective:**

To evaluate efficacy of multiple smoking cessation pharmacotherapy adaptations based on treatment response in Black adults who smoke daily.

**Design, Setting, and Participants:**

This randomized clinical trial of adapted therapy (ADT) or enhanced usual care (UC) included non-Hispanic Black adults who smoke and was conducted from May 2019 to January 2022 at a federally qualified health center in Kansas City, Missouri. Data analysis took place from March 2022 to January 2023.

**Interventions:**

Both groups received 18 weeks of pharmacotherapy with long-term follow-up through week 26. The ADT group consisted of 196 individuals who received a nicotine patch (NP) and up to 2 pharmacotherapy adaptations, with a first switch to varenicline at week 2 and, if needed, a second switch to bupropion plus NP (bupropion + NP) based on carbon monoxide (CO)-verified smoking status (CO ≥6 ppm) at week 6. The UC group consisted of 196 individuals who received NP throughout the duration of treatment.

**Main Outcomes and Measures:**

Anabasine-verified and anatabine-verified point-prevalence abstinence at week 12 (primary end point) and weeks 18 and 26 (secondary end points). The χ^2^ test was used to compare verified abstinence at week 12 (primary end point) and weeks 18 and 26 (secondary end points) between ADT and UC. A post hoc sensitivity analysis of smoking abstinence at week 12 was performed with multiple imputation using a monotone logistic regression with treatment and gender as covariates to impute the missing data.

**Results:**

Among 392 participants who were enrolled (mean [SD] age, 53 [11.6] years; 224 [57%] female; 186 [47%] ≤ 100% federal poverty level; mean [SD] 13 [12.4] cigarettes per day), 324 (83%) completed the trial. Overall, 196 individuals were randomized to each study group. Using intent-to-treat and imputing missing data as participants who smoke, verified 7-day abstinence was not significantly different by treatment group at 12 weeks (ADT: 34 of 196 [17.4%]; UC: 23 of 196 [11.7%]; odds ratio [OR], 1.58; 95% CI, 0.89-2.80; *P* = .12), 18 weeks (ADT: 32 of 196 [16.3%]; UC: 31 of 196 [15.8%]; OR, 1.04; 95% CI, 0.61-1.78; *P* = .89), and 26 weeks (ADT: 24 of 196 [12.2%]; UC: 26 of 196 [13.3%]; OR, 0.91; 95% CI, 0.50-1.65; *P* = .76). Of the ADT participants who received pharmacotherapy adaptations (135/188 [71.8%]), 11 of 135 (8.1%) were abstinent at week 12. Controlling for treatment, individuals who responded to treatment and had CO-verified abstinence at week 2 had 4.6 times greater odds of being abstinent at week 12 (37 of 129 [28.7%] abstinence) than those who did not respond to treatment (19 of 245 [7.8%] abstinence; OR; 4.6; 95% CI, 2.5-8.6; *P* < .001).

**Conclusions and Relevance:**

In this randomized clinical trial of adapted vs standard of care pharmacotherapy, adaptation to varenicline and/or bupropion + NP after failure of NP monotherapy did not significantly improve abstinence rates for Black adults who smoke relative to those who continued treatment with NP. Those who achieved abstinence in the first 2 weeks of the study were significantly more likely to achieve later abstinence, highlighting early treatment response as an important area for preemptive intervention.

**Trial Registration:**

ClinicalTrials.gov Identifier: NCT03897439

## Key Points

**Question:**

Does adapting to different smoking cessation medication early within a failed quit attempt lead to higher rates of abstinence than the standard of care, which continues them on the same medication, among Black adults who smoke daily and are interested in quitting?

**Findings:**

In this randomized clinical trial of 392 Black adults who smoke daily, adapting individuals to varenicline and/or bupropion in combination with a nicotine patch after failure of nicotine patch monotherapy did not significantly improve abstinence rates relative to standard of care treatment with nicotine patch (17.4% vs 11.7%).

**Meaning:**

These findings suggest that multiple pharmacotherapy adaptations do not substantially improve quit rates and support the current standard of care, which continues individuals who smoke on the same medication regardless of treatment response.

## Teaser

This randomized clinical trial evaluates the efficacy of multiple smoking cessation pharmacotherapy adaptations based on treatment response in Black adults who smoke daily at a health center in Kansas City, Missouri.

## Introduction

About 1 of every 7 non-Hispanic Black adults in the United States smoke.^1^ Smoking remains the leading cause of preventable disease and death for all people in the United States, including Black adults, and is the leading cause of cancer deaths.^2^ Although smoking prevalence among Black adults is similar to the US national average,^1^ Black adults tend to smoke fewer days per month and fewer cigarettes per day^3^ but bear a disproportionate share of tobacco-related disease.^2,4^ This makes the improvement of tobacco-related interventions for Black adults a national health priority.^5^

Early abstinence is an important but overlooked target for intervention. More than two-thirds of individuals who smoke and do not achieve abstinence within 4 weeks of initiating pharmacotherapy do not achieve abstinence at later time points.^6,7,8^ The current standard care for tobacco treatment recommends continuing an individual on pharmacotherapy for 8 to 12 weeks even if they continue to smoke, which is in direct contrast to treatment of other chronic diseases (eg, hypertension, diabetes) where altering medications to achieve desired outcomes are commonplace.^9,10,11,12,13^

Within tobacco dependence, existing studies suggest changing pharmacotherapy results in higher rates of abstinence for those who do not show initial treatment response.^14,15,16,17,18,19^ One key study assessed response to a nicotine patch (NP) after 1 week of precessation therapy and 1 week after the targeted quit date (TQD).^17^ Participants who responded (ie, quit) remained on the NP, while those who did not respond (ie, continued smoking) were switched to either continuation of NP (control condition), rescue treatment with bupropion + NP, or rescue treatment with varenicline. Findings suggested that early treatment of individuals who continued smoking and were switched to bupropion + NP or varenicline had abstinence rates that were almost twice as high as those who continued smoking and remained on NP at the end of treatment and at the 6-month follow-up. In a large prospective cohort of individuals who smoke and use smoking cessation medications across multiple quit attempts, individuals who switched to a different medication for each quit attempt had significantly higher abstinence rates relative to early and late users of medication or those who repeated the same medication across all quit attempts.^15^ Importantly, the act of switching drove better success regardless of the designated medication, which is consistent with other studies that have found that previous pharmacotherapy failure does not affect treatment outcomes.^20,21^

Prior studies have adapted pharmacotherapy only once and/or focused on adaptation distal to a failed quit attempt,^14,15,16,17,18,19^ despite evidence that adapting therapy early minimizes decreases in self-efficacy, enhances treatment engagement, and could improve treatment response.^17^ Furthermore, participants have been predominantly White adults who smoke. Prior research suggests that Black adults who smoke may have different smoking patterns and behaviors that could alter treatment response, including smoking more menthol cigarettes,^22^ smoking fewer cigarettes per day,^3^ and having different preferences for pharmacotherapy,^23,24^ making it inappropriate to extrapolate findings for treatment of Black adults.

This study is the first to examine if multiple pharmacotherapy adaptions proximal to a failed quit attempt (adapted therapy [ADT]) lead to higher rates of abstinence for Black adults who smoke compared with continuing with a single pharmacotherapy for the duration of treatment (usual care [UC]). While this treatment approach may benefit all adults who smoke, we focus on Black adults who smoke because they bear a disproportionate share of tobacco-related disease and have lower prevalence of pharmacotherapy supported sustained cessation relative to other racial or ethnic groups.^2,25^ Disparities are due to both the historical and present-day forms of systemic racism in the US, which have contributed to the overrepresentation of poverty and unequal access to work, education, housing, health insurance, and quality health care for Black US residents.^26,27,28,29,30^ Furthermore, racial bias and discriminatory practices in the US health care system, including exploitation of Black individuals, unequal treatment, and clinician bias contribute to lower likelihood of receiving clinician advice or assistance to quit^31^ and overall mistrust of recommended treatments.^23,32^ The primary hypothesis was that individuals randomized to the ADT group would have significantly higher abstinence at week 12 than participants randomized to UC. Secondary aims compared group differences in abstinence at weeks 18 and 26 and, in the ADT group, examined the proportion adapted at each time point and verified abstinence by path.

## Methods

### Trial Design

The study was a 26-week unblinded and open label randomized clinical trial to evaluate the efficacy of multiple pharmacotherapy adaptations in combination with counseling for smoking cessation among Black adults who smoke daily.^33^ Participants provided written informed consent. Study procedures were approved and monitored by the University of Kansas Medical Center institutional review board. Enrollment occurred between May 2019 and June 2021, with final follow-up in January 2022. This study followed the Consolidated Standards of Reporting Trials (CONSORT) reporting guideline. The trial protocol is included in Supplement 1.

### Participants

The study was conducted at Swope Health, a federally qualified health center in Kansas City, Missouri. Participants were recruited through in-clinic and community-based efforts in the metropolitan Kansas City area, including flyers; physician letters; direct referrals; health fairs; in-clinic recruitment; radio, television, and social media advertisements; and word-of-mouth referrals from current and former participants.^33^ Eligible participants self-identified as non-Hispanic African American or Black, were 18 years or older, smoked between 5 to 30 cigarettes per day for at least 25 of the past 30 days, registered an exhaled carbon monoxide (CO) of 5 ppm or higher at enrollment, and were interested in quitting smoking and willing to set a TQD within 7 days of enrollment. Exclusion criteria included medical contraindications for NP, varenicline, or bupropion, a pharmacotherapy-assisted quit attempt in the past 30 days, past 30-day use of noncigarette tobacco products including e-cigarettes, and unwillingness to refrain from the use of electronic cigarettes or nonstudy provided smoking cessation pharmacotherapy.

### Randomization

After providing written consent, participants were individually randomized 1:1 to ADT (n = 196) or UC (n = 196) groups. Randomization was stratified based on sex assigned at birth and baseline cigarettes per day smoked.

### Intervention

**Figure 1.**
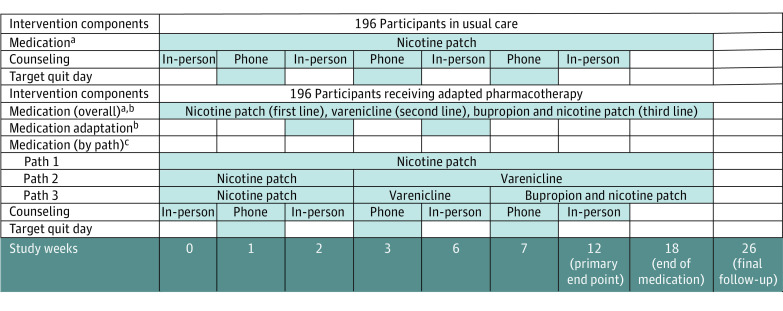
Intervention Activities by Treatment ^a^Medication was dispensed at weeks 0, 2, and 6. All participants started 2 weeks of nicotine patch and received 18 total weeks of pharmacotherapy during the study. ^b^Smoking status was monitored via exhaled carbon monoxide at weeks 2 and 6. Carbon monoxide of 6 ppm or higher was determined a priori to indicated continued smoking (ie, treatment nonresponse), which triggered adapation to the next pharmacotherapy. ^c^Carbon monoxide monitoring resulted in 3 possible treatment pathways: path 1, nicotine patch; path 2, varenicline; and path 3, bupropion + NP.

Treatment included 18 weeks of study medication (NP, varenicline, bupropion + NP), 7 sessions of counseling, and follow-up through week 26 (Figure 1). Participants were eligible to receive $350 in total compensation for completing study activities. Remuneration was based on visit attendance and not smoking status.

#### Pharmacotherapy

Participants randomized to UC received 18 weeks of 24-hour 21 mg NP. Participants randomized to ADT received 2 weeks of 24-hour 21 mg NP at baseline and up to 2 pharmacotherapy adaptations, with a first switch to varenicline and a second switch, if needed, to bupropion + NP based on CO-verified smoking status (CO ≥6 ppm) at weeks 2 and 6. Medications were dispensed at weeks 0, 2, and 6, with TQDs occurring at weeks 1, 3, and 7. The week 2 adaptation occurred after 2 weeks of patch therapy and 1 week after the first TQD. The week 6 adaptation occurred after 4 weeks of varenicline therapy and 3 weeks after the second TQD, allowing 1 week to titrate to the full dose of varenicline and 3 weeks of a quit attempt after reaching the full dose. Medication selection and order included NP because it is available over the counter and is the most used among Black adults who smoke,^24,34^ varenicline as the most effective medication,^35,36^ and bupropion + NP to reflect the benefit of combination therapies.^17,37^

ADT participants with a CO status of 5 ppm^38^ or less at follow-ups at weeks 2 and/or 6 were considered to have responded to treatment and were continued on their existing pharmacotherapy. Those with a CO status of 6 ppm or more were considered to have not responded to treatment and were switched to the next pharmacotherapy.

#### Counseling

Evidence-based, individualized, and culturally specific counseling^19,20^ was conducted in-person at weeks 0 (baseline), 2, 6, 12 and by phone at weeks 1, 3, and 7. Counseling focused on managing smoking cues, triggers, and the acute positive reinforcing effects of smoking in addition to dealing with nicotine withdrawal and craving.^35,39^ Counselors were accredited Tobacco Treatment Specialists supervised by a clinical psychologist (L.S.C.).

### Measures

Demographic and tobacco use history, adverse effects,^40,41,42,43^ counseling attendance, cessation fatigue (ie, “How tired are you of trying to quit smoking or stay quit today?”),^44^ commitment to abstinence (ie, “Which of the following best describes your current goal for quitting cigarettes over the next 2 weeks?” with response options of quit completely, cut back but not quit, and no longer interested in quitting),^45^ withdrawal,^46^ and craving^47^ were collected to quantify response to treatment. Medication adherence was assessed via 7-day Timeline Follow Back Interview at each in-person visit and corroborated with patch and pill counts.^48,49^ Adherence was defined as those who took 80% or more of the prescribed doses.

### Outcomes

The primary end point was anatabine- and anabasine-verified 7-day point prevalence smoking abstinence (≤2 ng/ml^50^) at week 12 using intent-to-treat principles and treating missing as participants who smoke. Secondary outcomes were anatabine and anabasine-verified 7-day point prevalence abstinence at week 18 (end-of-treatment) and 26 (follow-up). Week 12 was selected as the primary end point because of the focus on early treatment response and to allow time for all adaptations to occur.

### Sample Size

Postulated outcomes at week 12 were 18% abstinence among participants in UC^51,52,53^ and 32% abstinence among participants in ADT,^17,37^ treating those lost to follow-up as participants who smoke per the Russell standard.^54^ With 196 participants per group, the study provided 90% power to detect the expected differences with a type I error rate of 5%.

### Statistical Analysis

Participants were analyzed according to their randomized treatment condition. The χ^2^ test was used to compare verified abstinence at week 12 (primary end point) and weeks 18 and 26 (secondary end points) between ADT and UC. A post hoc sensitivity analysis of smoking abstinence at week 12 was performed with multiple imputation using a monotone logistic regression with treatment and sex as covariates to impute the missing data. Verified and self-reported abstinence for individuals who completed the study was also compared. Anatabine and anabasine are the reference standard for confirming abstinence in circumstances where cotinine measurements are invalid because detectable levels of cotinine could reflect nicotine replacement therapy use, smoking, or both.^55^ A cut-point of 2 ng/mL or less for both alkaloids was used to differentiate individuals who smoke from those who do not smoke.^50^ The study experienced less than 15% missing data at the week 12 primary end point and missingness was not related to group or baseline characteristics. χ^2^ and *t* tests were used to compared differences in treatment adherence, cessation fatigue, commitment to abstinence, and treatment-related side effects between groups. Post hoc logistic regression analyses modeled week 2 early treatment response as an estimator of week 12 abstinence. SAS, version 9.4M7 (SAS Institute), was used for all statistical analyses with *P* < .05 indicating statistical significance, and all tests were 2-tailed. Initial data analysis started in March 2022 and continued through January 2023.

## Results

**Figure 2.**
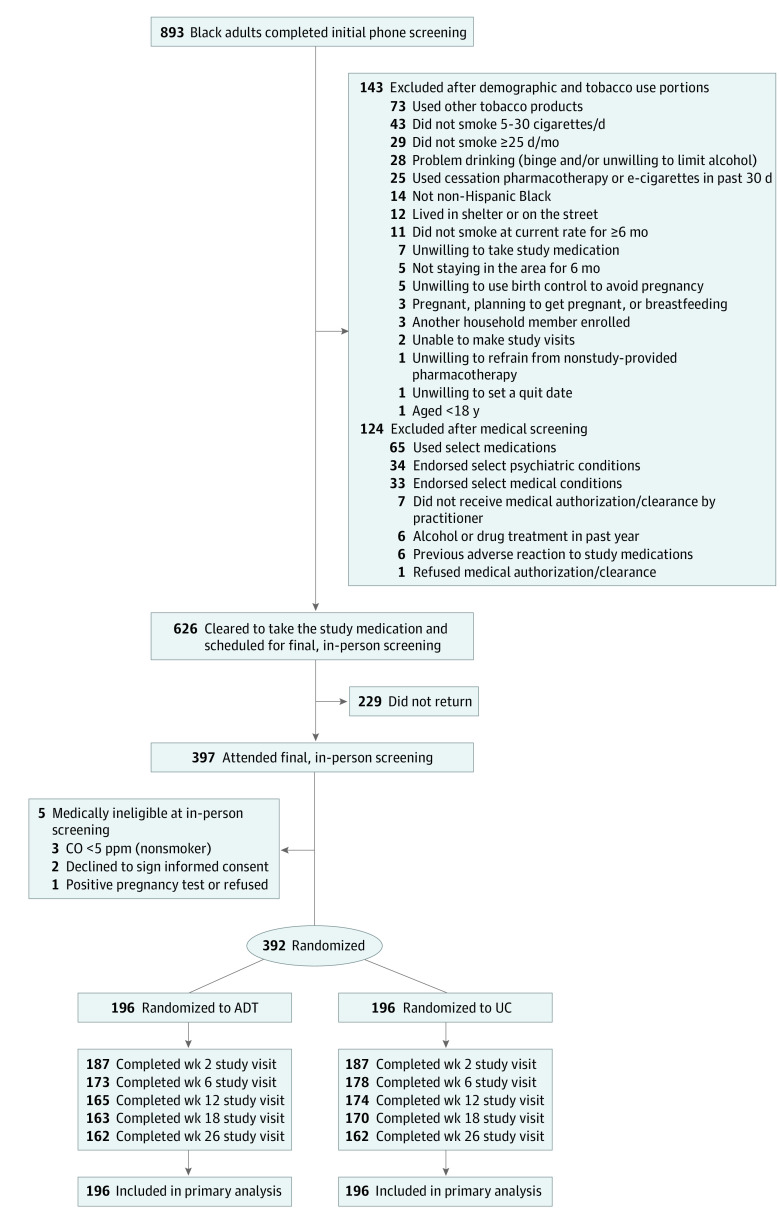
Study Flow Diagram ADT indicates adapted therapy; UC, usual care.

Among 392 participants (mean [SD] age, 53 [11.6] years; 224 [57%] female; 186 [47%] ≤ 100% federal poverty level; mean [SD] 13 [12.4] cigarettes per day), 324 (83%) completed the trial. Table 1 presents participant characteristics, and Figure 2 depicts enrollment and retention of participants. Retention was comparable between treatment groups; with 165 of 196 (84.5%) completing week 12 in the ADT group and 174 of 196 (88.8%) completing week 12 in the UC group (*P* = .18).

**Table 1.** Select Baseline Characteristics

Characteristics	Participants, No. (%)	
	Adapted group (n = 196)	Usual care group (n = 196)
Age, mean (SD), y	52.3 (11.7)	53.0 (11.5)
Sex assigned at birth		
Female	114 (58.2)	110 (56.1)
Male	82 (41.8)	86 (43.9)
Married or living with a partner	54 (27.5)	62 (31.6)
Employed	89 (45.4)	72 (36.7)
Some college or technical school	86 (43.9)	101 (51.5)
Had health insurance	124 (63.3)	138 (70.4)
Total gross household income		
Mean (SD), $	24 359.64 (19 834.43)	25 904.45 (21 083.24)
Median (IQR), $	17 000.00 (10 000.00-34 000.00)	20 000.00 (10 000.00-35 000.00)
≤100% Federal poverty level	99 (50.5)	87 (44.4)
Own a home	29 (14.8)	36 (18.4)
Age when participant started smoking regularly, mean (SD), y	18.1 (5.2)	18.6 (6.1)
Length of time as a person who smoked in years, mean (SD), y	34.1 (12.2)	34.4 (12.5)
Cigarettes per day in the past 7 d, mean (SD)	12.2 (6.9)	13.3 (7.3)
Smoke menthol cigarettes	173 (88.3)	172 (87.8)
Smoke within 30 min of waking^a^	163 (83.2)	153 (78.1)
No 24 h quit attempts in the past year	132 (67.4)	120 (61.2)
Moderate depressive symptoms^b^^,^^c^	43 (21.9)	35 (17.9)
>Moderate anxiety symptoms^c^^,^^d^	40 (20.4)	30 (15.3)
Marijuana use past 7 d	36 (18.4)	47 (24.0)
Urinary cotinine, mean (SD), ng/mL^e^	3012.8 (2474.6)	3360.6 (2656.4)
3-hydroxycotinine/cotinine, mean (SD), ng/mL^e^	3.4 (3.3)	3.5 (5.1)

^a^ Thirty minutes or less indicates clinically significant nicotine dependence.

^b^ Measured with the 9-Item Patient Health Questionnaire depression screening; range, 0-27 (most severe), scores of 10 or greater to indicate moderate.

^c^ Participants with a history of depression or anxiety with new or worsening symptoms in the last 6 months, treatment changes in the last 3 months, and those who did not feel that their symptoms were under control were excluded; participants with any suicide thoughts or attempts in past 6 months also excluded.

^d^ Measured with the 7-item Generalized Anxiety Disorder screening, range 0-21 (most severe), scores of 10 or greater to indicate moderate.

^e^ Values represent total (free/unconjugated plus glucuronide).

### Abstinence

Biochemically verified 7-day abstinence at weeks 12, 18, and 26 was not significantly different between treatment groups (Table 2). At week 12, 34 of 196 (17.4%) of ADT participants and 23 of 196 (11.7%) of UC participants were quit (odds ratio [OR], 1.58; 95% CI, 0.89-2.80; *P* = .12) based on intent-to-treat analysis with those missing imputed as adults who smoke. Week 18 (ADT: 32 of 196 [16.3%] vs UC: 31 of 196 [15.8%]; OR, 1.04; 95% CI, 0.61-1.78; *P* = .89) and week 26 (ADT: 24 of 196 [12.2%] vs UC: 26 of 196 [13.3%]; OR, 0.91; 95% CI, 0.50-1.65; *P* = .76) verified 7-day abstinence was also not significantly different between treatment groups based on intent-to-treat analysis with those missing imputed as participants who smoke. Analyses based on verified and self-reported abstinence among individuals who completed the study only provided similar results. Furthermore, treatment condition had no effect on withdrawal and cravings (eFigures 1 and 2 in Supplement 2).

**Table 2.** Biochemically Verified and Self-reported 7-Day PPA Rates by Treatment^a,b^

Smoking abstinence	Participant, No. (%)		OR (95% CI)	*P* value
	ADT group	UC group		
**7-d PPA verified with anatabine and anabasine: intent-to-treat with missing treated as adults who smoke^c^**				
Quit at week 12 (primary)	34/196 (17.4)	23/196 (11.7)	1.58 (0.89-2.80)	.12
Quit at week 18	32/196 (16.3)	31/196 (15.8)	1.04 (0.61-1.78)	.89
Quit at week 26	24/196 (12.2)	26/196 (13.3)	0.91 (0.50-1.65)	.76
**7-d PPA verified with anatabine and anabasine: completers only^a^**				
Quit at week 12	34/165 (20.6)	23/174 (13.2)	1.70 (0.96-3.04)	.07
Quit at week 18	32/163 (19.6)	31/170 (18.2)	1.10 (0.63-1.90)	.75
Quit at week 26	24/162 (14.8)	26/162 (16.1)	0.91 (0.50-1.66)	.76
**7-d PPA: self-reported completers only**				
Quit at week 12	60/165 (36.4)	50/174 (28.7)	1.42 (0.90-2.24)	.13
Quit at week 18	63/163 (38.7)	54/170 (31.8)	1.35 (0.86-2.13)	.19
Quit at week 26	55/162 (34.0)	56/162 (34.6)	0.97 (0.61-1.54)	.91

Abbreviations: ADT, adapted therapy; NP, nicotine patch; OR, odds ratio; PPA, point prevalence abstinence; UC, usual care.

^a^ Urine was collected by study staff at weeks 12, 18, and 26 to confirm 7-day self-reported abstinence from cigarettes via anatabine and anabasine. All but 4 participants who returned at week 12 (335/339), 2 (331/333) at week 18, and 2 (322/324) at week 26 provided urine samples for analysis.

^b^ Participants in both groups received 7 smoking cessation counseling sessions and an 18-week supply of smoking cessation pharmacotherapy. ADT participants received NP and up to 2 pharmacotherapy adaptations to varenicline and bupropion + NP based on CO-verified smoking status (CO ≥6 ppm) at weeks 2 and 6. UC participants received NP throughout the duration of treatment.

^c^ Participants lost to follow-up were imputed as individuals who smoke. Missingness was not related to group or baseline covariates.

### Adaptation

Table 3 shows the proportion of participants in the ADT group who were adapted and demonstrated verified abstinence by path (NP, varenicline, and bupropion + NP). Excluding those who responded to NP at both time points and did not require adaptation (n = 22), 11 verified individuals who quit smoking were rescued at week 12 by using adapted therapy (8.1%).

**Table 3.** Number and Percentage of Participants at Each Adaptation Time Point and Verified Abstinent at Week 12 by Treatment and Path

Study time point	Participants, No./total No. (%)	
	ADT	UC
Week 0^a^		
NP	196/196 (100)	196/196 (100)
Week 2^b^		
NP	61/187 (32.6)	187/187 (100)
Varenicline	126/187 (67.4)	NA
Week 6^c^		
NP	52/173 (30.1)	178/178 (100)
Varenicline	34/173 (19.6)	NA
Bupropion + NP	87/173 (50.3)	NA
Abstinent individuals at week 12^d^^,^^e^		
NP	23/165 (13.9)	23/174 (13.2)
Varenicline	9/165 (5.5)	NA
Bupropion + NP	2/165 (1.2)	NA
Overall	34/165 (20.6)	23/174 (13.2)

Abbreviations: ADT, adapted therapy; NA, not applicable; NP, nicotine patch; UC, usual care.

^a^ Randomization.

^b^ First adaptation time point.

^c^ Second adaptation time point.

^d^ Primary end point.

^e^ Week 12 data is showing the number abstinent by group, overall and within path of ADT.

### Treatment Adherence

There was no difference in counseling session attendance between treatment, with participants in the ADT and UC groups completing a mean (SD) 5.4 (1.3) and 5.6 (1.2) of 7 possible counseling sessions, respectively (*P* = .34). The proportion of participants taking 80% or more of the prescribed medication by treatment group and within ADT by path is shown in eTable 1 in Supplement 2. Overall adherence to 80% or more of the prescribed medication was 117 of 196 (59.7%) in the ADT group and 109 of 196 (55.6%) in the UC group (*P* = .41) (eTable 1 in Supplement 2).

### Experience With Quitting

Among participants who completed the study, there were no differences by treatment group or over time related to cessation fatigue or their commitment to abstinence. On the final TQD (week 7), 138 of 166 ADT participants (83.1%) and 158 of 177 UC participants (89.3%) remained committed to quitting completely during the next 2 weeks (*P* = .13). Further, the mean (SD) cessation fatigue scores at the primary end point (week 12) were indicative of being not at all tired of trying to quit or stay quit (ADT: 3.5 [4.3]; UC: 3.9 [4.3]; *P* = .33).

### Adverse Events

No group differences were observed in global experience of adverse effects through week 18, with 48 of 190 in the ADT group (25.3%) and 36 of 190 in the UC group (19.0%) experiencing any adverse effects (eTable 2 in Supplement 2). One death occurred but was unrelated to study participation.

### Early Treatment Response

Post hoc analyses of individuals who completed the study (n = 374) indicated that a similar proportion of those in ADT (61 of 187 [32.6%]) and UC (60 of 165 [36.4%]) had a CO score of 5 ppm or less at week 2 (*P* = .45), indicating that lack of a treatment effect was not due to better response to NP in the first 2 weeks in UC vs ADT. Imputing those who were missing at week 2 as early treatment individuals who did not respond to treatment and imputing those lost to follow-up at week 12 as adults who smoke (n = 392), treatment response based on CO-verified abstinence at week 2 (OR, 5.0; 95% CI, 2.7-9.1; *P* < .001) but not treatment (OR, 1.7; 95% CI, 0.9-3.1; *P* = .09) estimated week 12 abstinence. Analyses including only participants who completed the study revealed similar findings. Of participants who demonstrated early treatment response at week 2, 37 of 129 (28.7%) were verified abstinent at week 12 compared with 19 of 245 (7.8%) abstinent among participants who did not respond to early treatment (OR; 4.6; 95% CI, 2.5-8.6; *P* < .001).

## Discussion

Adaptation to varenicline and/or bupropion + NP after the failure of NP monotherapy did not significantly improve abstinence rates for Black adults who smoke relative to continued treatment with NP. The lack of treatment association was not explained by group differences in interest of quitting at study onset, early response to NP, or cessation fatigue.

The lack of treatment association is largely explained by a lower-than-expected effect of adapted therapy, with ADT rescuing only 5% of adults who smoke as opposed to the 15% anticipated. That UC worked as well as ADT is encouraging given that UC requires fewer components, is less costly, and is easier to manage within the health care system. Our findings add to a growing body of literature suggesting that multicomponent interventions increase participant burden and decrease adherence without conferring additional treatment benefit and lead to lower rates of abstinence than interventions with fewer components.^14,19,56,57^ In the current study, adherence to new pharmacotherapy adaptations was low and resulted in only 11 additional participants who quit.

The lack of treatment association is also explained by the fact that nearly one-third of those in ADT never required adaptation (ie, remained on NP monotherapy). Participants who responded to NP during the first 2 weeks had 5 times greater odds of achieving abstinence at the primary end point relative to those who did not respond. This finding is consistent with the tipping point hypothesis, which suggests that individuals close to a hypothetical tipping point benefit greatly from limited and varied interventions, while those far from the tipping point benefit very little from even a strong intervention.^56^ Defining the hypothetical tipping point is important for clinical practice. Previous studies have suggested that gender, successful prior abstinence from smoking of more than 1 month, baseline nicotine dependence, and the number of days abstinent in the first 2 weeks of treatment are important tipping point factors.^56^ Future research will determine how these factors and others (eg, marijuana and other tobacco product use, ongoing mental health concerns, socioeconomic disadvantage) distinguish individuals who respond to early treatment responders from those who do not respond. Characterizing individuals according to their likelihood of cessation success at treatment outset would allow better targeting of low-cost, widely available interventions, like NP, to individuals with a high likelihood of benefitting from any intervention. Finding efficacious approaches for individuals at high risk for cessation failure who are likely to confer only modest benefits from very intensive intervention remains an important area for future study.

The quit rates achieved overall were lower than projected and lower than those achieved by Black adults who smoke and were using NP patch therapy in similar studies,^36,51,52,53^ but reflect national trends from population-level data showing, after decades of progress, rates of quitting have stalled among individuals from underrepresented racial and ethnic groups and adults with low income who smoke despite high motivation to quit and more past-year attempts.^25^ Among Black adults who smoke in a recent population-level study,^25^ 78.8% were interested in quitting smoking in 2018 and 2019, 55.8% made a past year quit attempt, 23.2% with nicotine replacement therapy, 8.7% with varenicline or bupropion, and 1.5% to 5.9% with behavioral support, yet only 5.2% achieved cessation. Efforts to address unmet social needs among Black adults who smoke and adults with low income who smoke and a focus on tobacco harm reduction among people who smoke and are unwilling or unable to quit have shown promise and are important areas for future study.^58,59,60,61,62,63^

### Limitations

This study had limitations. Our study was restricted to Black adults who smoke daily and are interested in stopping smoking. The UC intervention was more intense than treatment typically provided in primary care but is consistent with recommended practice.^35^ Because this enhanced UC treatment served as a more rigorous comparator, quit rates achieved in the UC group are likely higher than those expected with standard advice to quit accompanied by treatment with nicotine replacement. The 3-weeks of full dose varenicline prior to the switch to bupropion + NP may have been insufficient to yield varenicline’s full effect given evidence that some individuals quit late in a quit attempt with varenicline.^55^ Although, even among those who stayed on varenicline from week 2 through week 18, quitting was low overall. Adherence to varenicline and bupropion + NP was lower than mono-NP; however, determining if the medication, the order in which it was prescribed, the process of adaptation, or other factors were associated with medication adherence are beyond the scope of this study. Findings reflect use of medication following adaptation and are relevant to clinical practice.

## Conclusions

In this randomized clinical trial of adapted pharmacotherapy vs standard of care pharmacotherapy, adaptation to varenicline and/or bupropion + NP after failure of NP monotherapy did not significantly improve abstinence rates for Black adults who smoke relative to continued treatment with NP. However, these findings suggest that those who responded to pharmacotherapy in the first 2 weeks of the study had a 5 times greater odds of being abstinent at week 12. Findings highlight the continued need to identify effective treatment, particularly for those at high risk for cessation failure and those disproportionately impacted by tobacco-related disease.

## Notes

Supplement 1.
Trial Protocol

Supplement 2. **eFigure 1.** 10-Item Brief Questionnaire of Smoking Urges **eFigure 2.** 8-Item Minnesota Tobacco Withdrawal Scale **eTable 1.** Medication Compliance of 80% or More by Treatment and Timepoint in Those Who Returned at Each Time Point **eTable 2.** Prevalence of Treatment-Related Adverse Effects by Symptom and Globally for Participants in ADT and UC

Supplement 3.
Data Sharing Statement
