# Supplementary material for: Multiple Pharmacotherapy Adaptations for Smoking Cessation Based on Treatment Response in Black Adults Who Smoke: A Randomized Clinical Trial
Source: JAMA Netw Open. 2023 Jun 20;6(6):e2317895. doi: 10.1001/jamanetworkopen.2023.17895 (PMC10282892; doi:10.1001/jamanetworkopen.2023.17895)
Supplement: Supplement 2. — eFigure 1. 10-Item Brief Questionnaire of Smoking Urges eFigure 2. 8-Item Minnesota Tobacco Withdrawal Scale eTable 1. Medication Compliance of 80% or More by Treatment and Timepoint in Those Who Returned at Each Time Point eTable 2. Prevalence of Treatment-Related Adverse Effects by Symptom and Globally for Participants in ADT and UC [file jamanetwopen-e2317895-s002.pdf]

Supplemental Online Content

Nollen NL, Ahluwalia JS, Mayo MS, et al. Multiple pharmacotherapy adaptations for smoking cessation based on treatment response in Black adults who smoke: a randomized clinical trial. *JAMA Netw Open*. 2023;6(6):e2317895. doi:10.1001/jamanetworkopen.2023.17895

eFigure 1. 10-Item Brief Questionnaire of Smoking Urges

eFigure 2. 8-Item Minnesota Tobacco Withdrawal Scale

eTable 1. Medication Compliance of 80% or More by Treatment and Timepoint in Those Who Returned at Each Time Point

eTable 2. Prevalence of Treatment-Related Side Effects by Symptom and Globally for Participants in ADT and UC

This supplemental material has been provided by the authors to give readers additional information about their work.

© 2023 Nollen NL et al. *JAMA Network Open.*

eFigure 1. 10-Item Brief Questionnaire of Smoking Urges

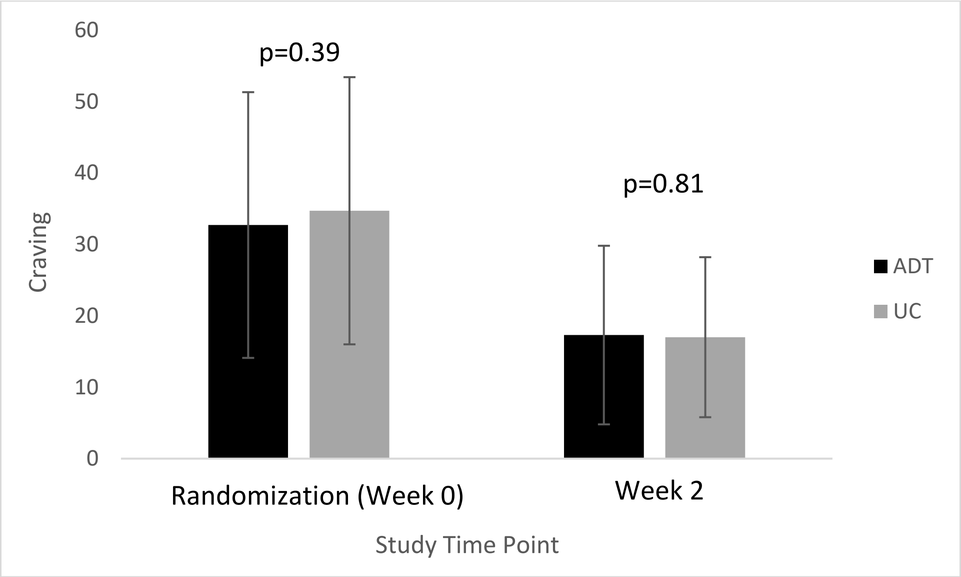

The 10-item Brief Questionnaire of Smoking Urges assessed smoking-related craving.[50] Total scores are presented by treatment group at randomization (week 0) and week 2. Scores can range from 10-70 with higher scores indicating greater overall craving. ADT=Adaptive Therapy, UC=Usual Care. Error bars represent standard deviations.

eFigure 2. 8-Item Minnesota Tobacco Withdrawal Scale

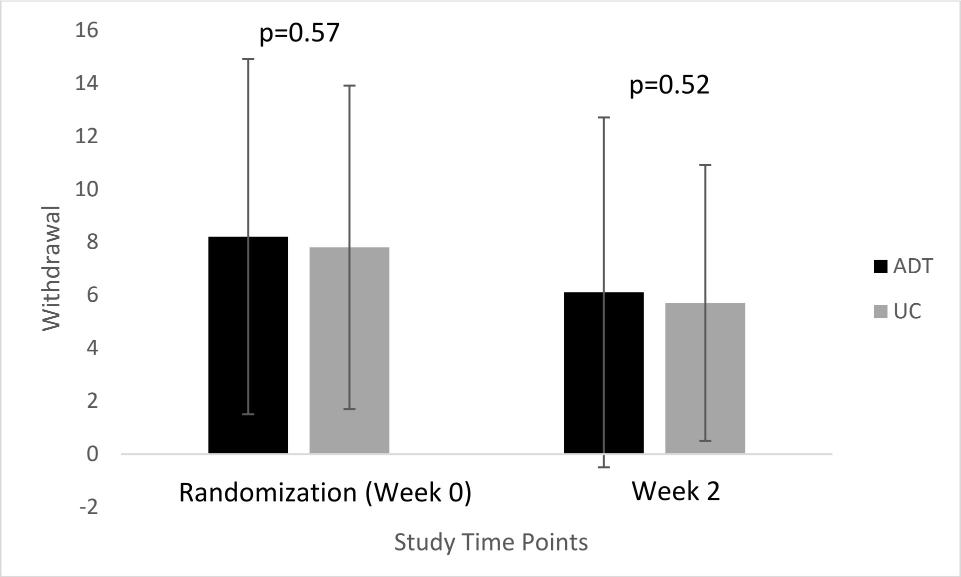

The 8-item Minnesota Tobacco Withdrawal Scale assessed common symptoms of withdrawal including anxiety, depressed mood, difficulty concentrating, irritability, increased appetite, insomnia, restless, desire to smoke. [49] Total scores are presented by treatment group at randomization (week 0) and week 2. Scores can range from 0-32 with higher scores indication greater overall withdrawal. ADT=Adaptive Therapy, UC=Usual Care. Error bars represent standard deviations.

© 2023 Nollen NL et al. *JAMA Network Open.*

eTable 1. Medication Compliance of 80% or More by Treatment and Timepoint in Those Who Returned at Each Time Point[a,b]

	No (%)						
	Overall[c]	Week 2	**Week 6**		Week 12		
		Patch	Patch	Varenicline	Patch	Varenicline	Bupropion
**ADT (n=196))**							
	117/196 (59.7%)	--	--	--	--	--	--
NP (n=53)	--	38/52 (73.1%)	41/51 (80.4%)	--	39/51 (76.5%)	--	--
VAR (n=48)	--	28/47 (59.6%)	--	17/31 (54.8%)	--	17/29 (58.6%)	--
BUP+NP (n=87)	--	52/85 (61.2%)	--	46/83 (55.4%)	44//76 (57.9%)	--	21/76 (27.6%)
**UC (n=196)**							
	109/196 (55.6%)	117/183 (63.9%)	134/177 (75.7%)	--	114/166 (68.7%)	--	--

[a]Participants reported use of medication each day for the last 7 days at weeks 2, 6, 12, and 18. Compliance was defined as ≥ 80% of medication taken out of medication prescribed.
[b]Eight participants (4.1%) in OPT were missing and never adapted
[c]Represented as the proportion achieving ≥ 80% compliance at the week 2, 6, and 12 visits. Participants who were missing were counted as non-compliant, p=0.41

© 2023 Nollen NL et al. *JAMA Network Open.*

eTable 2. Prevalence of Treatment-Related Side Effects by Symptom and Globally for Participants in ADT and UC

	No. (%)	
	By Symptom,[b] Weeks 2,6,12,18	
	ADT<br>n=190	UC<br>n=190
Bowel changes like constipation or diarrhea	6 (3.2)	2 (1.1)
Breathing problems	6 (3.2)	2 (1.1)
Cold sweats or increased sweating	2 (1.1)	0
Difficulty concentrating or confusion/mental weakness	2 (1.1)	1 (0.5)
Disturbed hearing/vision	0	0
Dizziness	8 (4.2)	2 (1.1)
Dry Mouth	5 (2.6)	0
Gas	1 (0.5)	1 (0.5)
Headache	9 (4.7)	3 (1.6)
Hostility, aggression, or other change in behavior not normal for you	2 (1.1)	0
Irregular or fast heartbeat	1 (0.5)	1 (0.5)
Irritability	3 (1.6)	0
Nausea or vomiting	14 (7.4)	5 (2.6)
Nervous/restless/anxious	8 (4.2)	5 (2.6)
Redness/swelling/rash/irritation on the skin	19 (10.0)	25 (13.2)
Sleeping problems	14 (7.4)	3 (1.6)
Stomach cramps or pains	3 (1.6)	2 (1.1)
Tiredness/fatigue	4 (2.1)	2 (1.1)
	Global, Weeks 2,6,12,18	
	ADT	UC
Any[c]	48 (25.3)	36 (19.0)
Serious Adverse Events (SAE)[d]	1	0

[a]ADT treatment began at Week 2 and continued through Week 18. 6 in ADT and 6 in UC did not return from weeks 2, 6, 12 or 18 and were excluded from these analyses. All associated p values were calculated with Chi-squared test or Fisher's Exact test where appropriate.[b] No. and % of participants that reported experiencing each symptom (yes, no) between Weeks 2-18. [c]No. and % of participants that reported any of the above symptom between Weeks 2-18.

[d] Serious Adverse Events: one participant passed away prior to completing week 26. The death occurred 41 days past the end-of-treatment, and they had returned study medications. The death was determined to be unrelated to the study by the medical monitor.

© 2023 Nollen NL et al. *JAMA Network Open.*
