# Supplementary material for: Multiple Pharmacotherapy Adaptations for Smoking Cessation Based on Treatment Response in Black Adults Who Smoke: A Randomized Clinical Trial
Source: JAMA Netw Open. 2023 Jun 20;6(6):e2317895. doi: 10.1001/jamanetworkopen.2023.17895 (PMC10282892; doi:10.1001/jamanetworkopen.2023.17895)
Supplement: Supplement 3. — Data Sharing Statement [file jamanetwopen-e2317895-s003.pdf]

Data Sharing Statement

Nollen. Multiple Pharmacotherapy Adaptations for Smoking Cessation Based on Treatment Response in Black Adults Who Smoke. *JAMA Netw Open*. Published June 20, 2023. doi:10.1001/jamanetworkopen.2023.17895

Data
Data available: Yes
Data types: Deidentified participant data, Data dictionary
How to access data: Available upon request to nnollen@kumc.edu
When available: With publication

Supporting Documents
Document types: Informed consent form
How to access documents: Available upon request to nnollen@kumc.edu
When available: With publication

Additional Information
Who can access the data: Researchers whose proposed use of the data has been approved
Types of analyses: For a specified purpose that has been approved
Mechanisms of data availability: With a signed data access agreement
